# Supplementary material for: Acute Aquatic Toxicity to Zebrafish and Bioaccumulation in Marine Mussels of Antimony Tin Oxide Nanoparticles
Source: Nanomaterials (Basel). 2023 Jul 20;13(14):2112. doi: 10.3390/nano13142112 (PMC10385626; doi:10.3390/nano13142112)
Supplement: Supplementary file 1 [file nanomaterials-13-02112-s001.zip › nanomaterials-2431995-supplementary.pdf]

Supporting information

Acute Aquatic Toxicity to Zebrafish and Bioaccumulation in Marine Mussels of Antimony Tin Oxide Nanoparticles

Ivone Pinheiro[1], Monica Quarato[1], Antonio Moreda-Piñeiro[2], Ana Vieira[1], Virginie Serin[3], David Neumeyer[3], Nicolas Ratel-Ramond[3], Sébastien Joulié[3], Alain Claverie[3], Miguel Spuch-Calvar[4], Miguel A. Correa-Duarte[4], Alexandre Campos[5], José Carlos Martins[3], Pilar Bermejo-Barrera[2], Marisa P. Sarriá[1‡], Laura Rodriguez-Lorenzo[1], Begoña Espiña[1,]*

[1] Water Quality Group, INL-International Iberian Nanotechnology Laboratory, Av. Mestre José Veiga, 4715-330 Braga, Portugal
[2] Department of Analytical Chemistry , Nutrition and Bromatology, Faculty of Chemistry, University of Santiago de Compostela , Santiago de Compostela , Spain.
[3] CEMES/CNRS, 29, rue Jeanne Marvig, 31055 Toulouse, France
[4]TeamNanoTech / Magnetic Materials Group. CINBIO. Universidade de Vigo - Campus Universitario Lagoas Marcosende 36310, Vigo, Spain.
[5] CIIMAR - Interdisciplinary Centre of Marine and Environmental Research, 4450-208 Matosinhos, Portugal
‡ *current address*: European Commission, Joint Research Centre (JRC), Ispra, Italy.

E-mail: begona.espina@inl.int

Keywords

Antimony Tin Oxide Nanoparticles; bioaccumulation; zebrafish embryo; marine mussels; aquatic toxicity

Table of Contents

Materials and Methods 3

Mussel′ tissue sampling for transmission electron microscopy 3

Operating conditions for SP-ICP-MS measurements 3

Characterization of nanoparticles using X-ray diffraction 4

Results 5

Acute adult fish toxicity test 5

Acute fish embryo toxicity test 6

Sn and Sb ionic recoveries 7

Biodistribution of ATO NPs in tissues 8

Materials and Methods

Mussel' tissue sampling for transmission electron microscopy

After 28 days of exposure, one mussel from two of the three replica were used to collect fragments of mantel and digestive gland. The fragments were fixed overnight at 4 °C in a mixture of 2% (v/v) paraformaldehyde and 2.5% (v/v) glutaraldehyde in 0.1 M sodium cacodylate buffer, followed by a routine processing for transmission electron microscopy (TEM). Briefly, the fragments were post-fixed in a 1% (v/v) osmium tetroxide solution, dehydrated with increasing ethanol (from 50-100%) and with a final emersion on propylene oxide. The infiltration was made in mixtures of propylene oxide:epoxy resin (EMBed-812 kit) at different proportion, increasing the amount of resin to finally have just epoxy resin. The cure of the blocks was performed at 60 °C during three days.

Ultrathin sections (≈80 nm thick) were cut in a PowerTome PC ultramicrotome (RMC Boeckeler,USA), with a diamond knife (Diatome) and placed on copper formvar/carbon 200 grids. The micrographs of the tissue' sections were acquired using a JEOL JEM 1010 TEM operating at 100 kV.

Table S1. Operating conditions for sp-ICP-MS measurements.

Parameter (units)	
Instrument	NexIon 2000
Spray chamber	QuartzCyclonic
PC^{3x} Peltier Cooler System	4°C
Nebulizer type	PFA MicroFlow
RF power (W)	1600
Plasma gas flow rate (L min^{-1})	15

Auxiliary gas flow rate (L min^{-1})	1.2
Nebulizer gas flow rate (L min^{-1})	1.14
NH_3 flow rate (mL min^{-1})	0.30
Sample flow rate (mL min^{-1})	≈ 0.21
Quadrupole ion deflector (V)	Set for maximum ion transmission
Transport efficiency (%)	≈ 10%
Scan time (s)	60
Dwell time (µs)	50
RPq	0.35
Sn m/z	120
Sn mass fraction (%)	67

Characterization of nanoparticles using X-ray diffraction

X-ray diffraction measurements were performed on Bruker D8 Advance diffractometer, equipped with a Cu anticathode, programmable divergence slits, soller slits on primary and secondary arms, and a Lynxeye position sensitive detector (Bragg Brentano geometry).

The diffracted intensity was recorded in a 2theta range between 10 and 80°, with a step of 0.01° and an acquisition time of 1sec/step. The opening of the primary divergence slits was fixed at 0.3° during the acquisition.

In the case of the nanoparticles suspensions, the specimen was first dried in order to obtain a powder, with which the sample holder was filled.

Analysis of instrument broadening and calibrations of the instrument (definition of zero-offset, sample displacement) were performed using a standard LaB6 powder specimen provided by NIST (NIST SRM 660b). The measurement of the LaB6 powder was then treated using Rietveld refinement (implemented in TOPAS software), in order to calibrate the instrument broadening defined by the Caglioti parameters (U, V, W, X, Y, Z). These parameters were then used in the treatment of the specimen of

interest, in order to evaluate the crystallite size (when applicable), using a pattern matching approach

Results

Fish Acute Toxicity (FAT) test

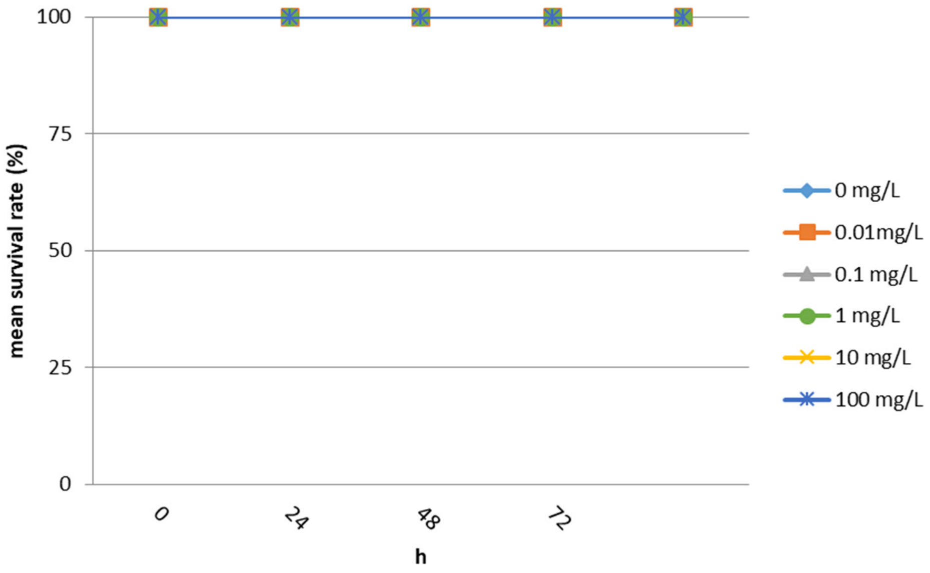

Figure S1: Results of FAT upon exposure to 0, 0.01, 0.1, 1, 10 and 100 mg/ L of ATO NPs, for 96 h.

Fish Embryo Acute Toxicity (FET) test

Table S2: Statistics of FET upon exposure to 0, 0.01, 0.1, 1, 10, and 100 mg /L of ATO NPs, for 80 h_{pf}. Effects on characteristic embryonic developmental stage-correspondent events are listed, and referred to "non-significant" (-) or "significant" (+) statistical differences among groups on tested independent variables.

	h_{pf}	independent variables	statistical analysis	ATO NPs	Effect
**Morphometric Analysis**	8	*epipolic arc*	One-Factor ANOVA	*F(5, 109)=0.256, P=0.936*	-
	8	*yolk volume*	ANCOVA	*F(5, 207)=0.835, P=0.526*	-
	32	*head-trunk angle*	One-Factor ANOVA	*F(5, 71)=1.320, P=0.266*	-
	56	*pupil surface*	ANCOVA	*F(5, 83)=0.916, P=0.475*	-
**Neuro-Mediated Traits**	32	*spontaneous movements*	Chi-Square test	χ^2= 22.952, *DF*= 5, *P*<0.050	+
	32	*heart rate*	One-Factor ANOVA	*F(5, 54)=3.813, P*<0.050	+
	56	*heart rate*	Nested ANOVA	*F(6, 108)=54.892, P<0.053*	-
	56	*hatching rate*	Chi-Square test	χ^2=4.026, *DF*=5, *P*=0.546	-
	80	*free-swimming*			
	80	*cumulative survival*	Chi-Square test	χ^2= 7.409 *DF*=5 *P*=0.192	-

Sn and Sb ionic recoveries

A

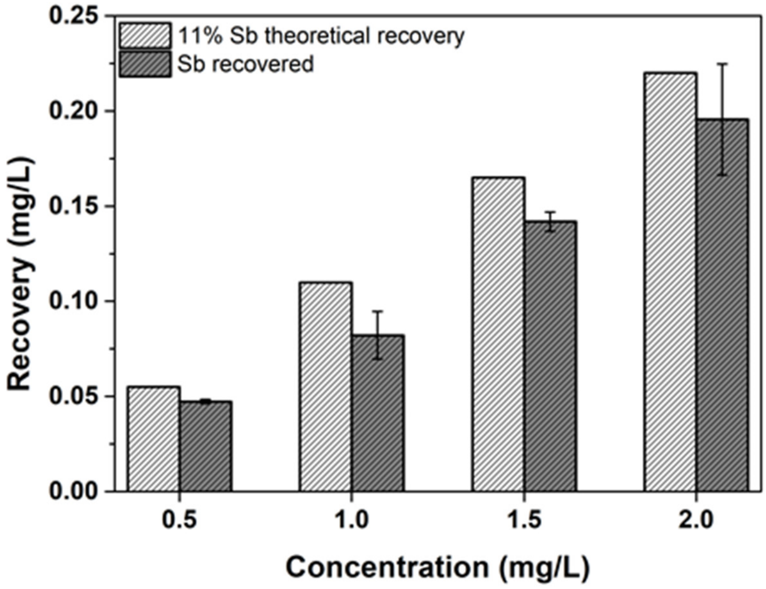

B

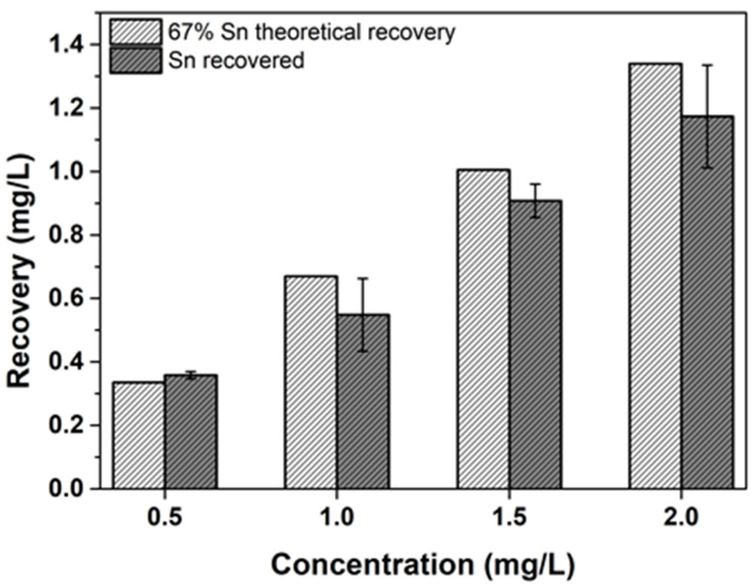

Figure S2. Dissolved concentrations of Sb (A) and Sn (B), recovered after mussels' alkaline digestion of spiked samples at different concentrations.

Biodistribution of ATO NPs in tissues

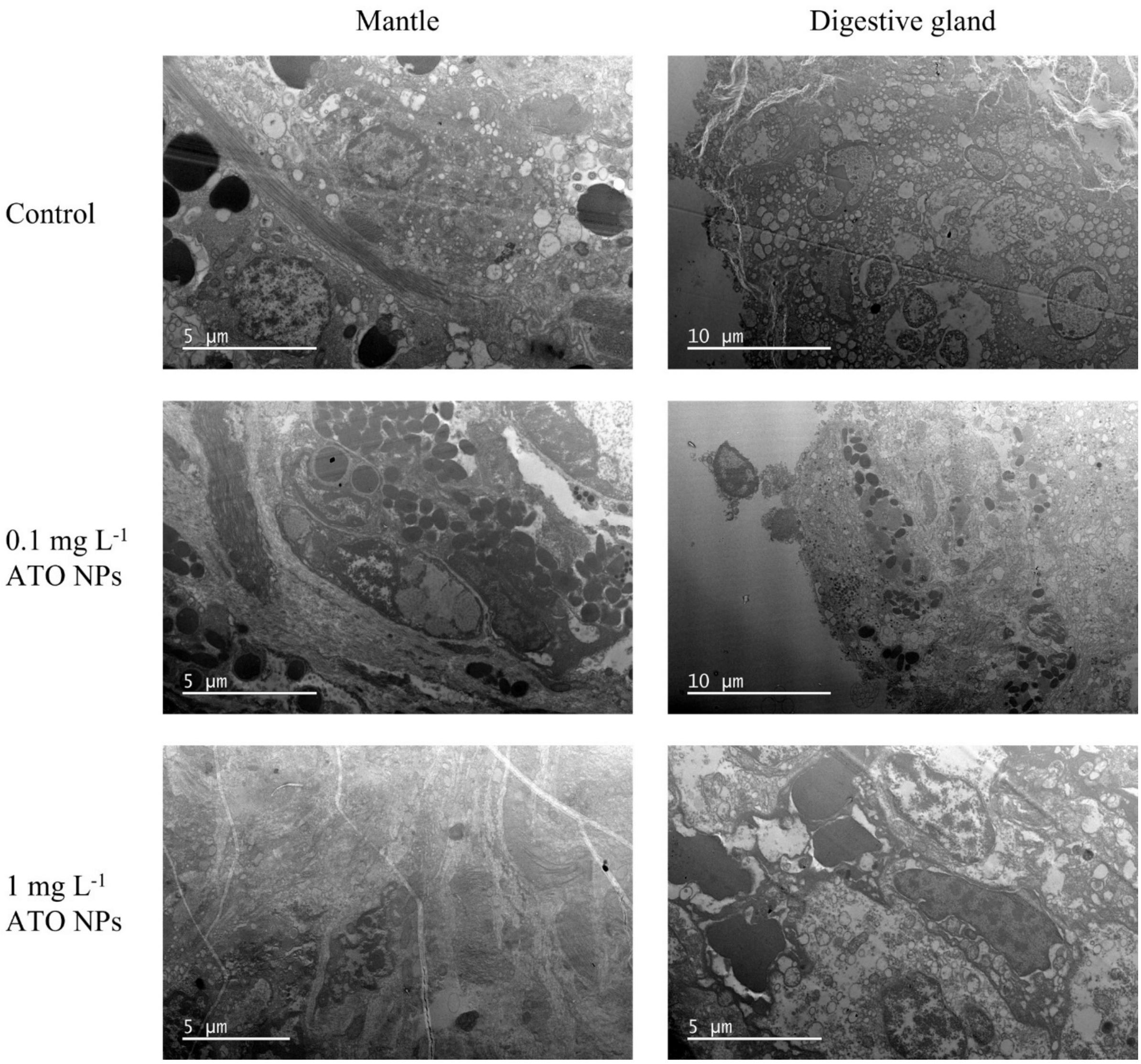

Figure S3: Transmission electron microscopy images of mussel' tissues (mantle, left panel; digestive gland, right panel) upon dietary exposure to 0 (top row), 0.1 (middle row) and 1 (bottom row) mg L^{-1} of ATO NPs, every 7 days, during 28 days. A normal tissue patterning was observed, and no evident effect of the nanoparticles was evidenced in the tissues ultrastructure.
